# Modelling Thermal Conduction in Nanoparticle Aggregates in the Presence of Surfactants

**DOI:** 10.3390/nano10112288

**Published:** 2020-11-19

**Authors:** Nikolaos P. Karagiannakis, Eugene D. Skouras, Vasilis N. Burganos

**Affiliations:** 1 Institute of Chemical Engineering Sciences (ICE-HT), Foundation for Research and Technology, Hellas (FORTH), GR-26504 Patras, Greece; nick_karag@iceht.forth.gr (N.P.K.); Eugene.Skouras@iceht.forth.gr (E.D.S.); 2 Department of Chemical Engineering, University of Patras, GR-26504 Patras, Greece; 3 Department of Mechanical Engineering, University of the Peloponnese, GR-26334 Patras, Greece

**Keywords:** nanofluid, heat conduction, effective thermal conductivity, particle aggregates, surfactants

## Abstract

Many theoretical and experimental studies have shown that the addition of nanoparticles into conventional fluids may generate nanofluids with significantly improved heat transfer properties. In the present work, the effect of nanoparticle aggregation on the thermal conductivity of nanofluids is studied, considering also the effect of surfactants that are typically added to stabilise the nanofluid. A method for simulating aggregate formation is developed here that allows tailoring of the fractal dimension and the number density of the nanoparticles to desired values. The method is shown to be computationally simple and fast. Data that are extracted from electron microscope images are compared with simulation results regarding surface porosity and the autocorrelation function. The surfactants are modelled as a layer around the particles, and the effective thermal conductivity is calculated with a meshless numerical technique. Significant increase in conductivity is observed for small values of the fractal dimension and for large number density of particles in the aggregate. The simulations are in good agreement with experimental results. It is also concluded that prediction of the conductivity of such nanofluids requires the knowledge of the type and the amount of the surfactant added.

## 1. Introduction

The term “nanofluids” was first introduced in 1995 [1] and defines a new class of fluids, which are created by the dispersion and suspension of particles smaller than 100 nm. The nanofluids are suspensions with comparative advantages, such as higher thermal conductivity, greater stability, and reduced corrosion. Due to their small size, nanoparticles have the ability to circulate smoothly in channels and pores of micrometre diameter, thus, minimising pore blocking, while their size prevents their sedimentation, making the suspensions more stable. Based on such advantages, nanofluids can be used in a variety of applications. Their use would allow for smaller and lighter machines, pumps and refrigerators, hence improving heating and cooling systems [2,3]. From experimental measurements made for the heating of buildings using nanofluids, it has been found that their application can significantly reduce energy requirements.

In addition, nanoparticles dispersed into bioliquids could be used in medicine as transport vehicles either for drugs, or for radiation, thus offering new treatment techniques. The application of nanofluids is already being considered for its safety and efficiency in nuclear reactors. Moreover, in the case of renewable energy sources nanofluids can be used to improve heat transfer from solar panels to heat storage tanks. Applications can be found even in the space sector, where similar devices develop very high amounts of heat.

In view of the theoretical study of nanoparticles behaviour, a large number of models have been developed in order to predict their properties. A classification of these models can be performed based on the main mechanism taken into account for the calculation of the thermal conductivity. Based on this criterion, they are divided into models based on the classical effective-medium theory, the existence of a nanolayer, the effect of Brownian motion [4,5], and the aggregation mechanism [6,7,8].

Brownian motion, also called thermal motion, is the result of various forces and is believed to affect indirectly the formation of aggregates, the presence of which significantly improves the thermal conductivity of nanofluids. Recent experimental studies [9,10,11,12,13] argue that when nanoparticles accumulate in small aggregates, they lead to a large increase in the effective conductivity. Additionally, a large increase in effective thermal conductivity is accompanied by a large increase in viscosity, which is indicative of the effect of aggregation [13]. Furthermore, some studies conclude that nanoparticles showing good dispersion do not present an unusual increase in thermal conductivity [14,15,16].

Nanocolloid aggregates are expected to transfer heat more efficiently compared to fully dispersed particles of the same volume fraction, due to the increased contact of the particles with each other and with the walls. Considering the nanofluid as a sum of aggregates and taking into account the fractal dimension of each nanofluid, efforts were made to predict the thermal conductivity [9].

On the other hand, larger mass aggregates can block heat transfer or cause sedimentation. As a result, aggregates can have a positive or a negative effect on the thermal conductivity of nanofluids, depending on the conditions. It is generally considered that the aggregation of nanoparticles is more likely to occur in nanofluids with a larger volume fraction due to the reduced distance between the particles which, in this case, increases the probability of aggregation. Particle clusters can result from collisions between nanoparticles. The aggregation and dispersion of particles, as well as the formation of clusters, are controlled by a variety of external and internal forces between the base fluid and the nanoparticles, as well as between the nanoparticles themselves [9,10,17].

Various predictive models have been proposed in the literature that attempt to estimate the thermal conductivity of aggregates. Evans [18] developed a three-level homogenisation model to predict the thermal conductivity of nanofluids based on the morphology of the aggregates. Another study [19] presents a prediction model for the calculation of the thermal conductivity of aggregates, extending Maxwell’s theory to non-spherical particles. Aggregates are treated as spheres with effective thermal conductivity and effective volume fraction. That study suggests that fractal structures can be represented by a single parameter, and presents a method for calculating this parameter by numerical simulations.

At the same time, a great number of models are based on the hypothesis of the presence of a nanostructure of a certain type around the nanoparticles [20,21]. The fluid molecules that are close to the nanoparticles can form an ordered layer, an almost solid structure, called a nanolayer. This layer can act as a thermal bridge between a solid nanoparticle and the liquid medium, leading to an increase in the thermal conductivity of the nanoparticle.

One of the first models based on the nanolayer approach, which aims to calculate the thermal conductivity of nanoparticles, is a modification of Maxwell’s model [20]. Maxwell’s model accurately predicts the conductivity of spherical particles suspensions, in the absence of thermal interactions among them, and is expressed as

(1) $$k_{eff}=\frac{k_{p}+2k_{f}+2\left(k_{p}-k_{f}\right)f_{p}}{k_{p}+2k_{f}-2\left(k_{p}-k_{f}\right)f_{p}}k_{f},$$

where $k_{p}$ is the conductivity of the particles, $k_{f}$ is the conductivity of the base fluid, and $f_{p}$ is the volume fraction of the particles. In order to include the effect of the nanolayer on the model, four assumptions were made [20]. First, the molecules within the nanolayer are more ordered than those of the liquid medium. Second, the thermal conductivity of the ordered layer $k_{l\;}$is much greater than that of the liquid medium. Third, the nanolayer can be combined with the particle to form an effective, thermally equivalent particle. Finally, the volume fraction of the particles is so small that there is no overlap between the effective particles.

Assuming a layer of thickness δ, the volume fraction of the effective particle will be $f_{pe}=f_{p}{\left(1+\beta \right)}^{3}$, where $\beta =\delta /r_{p}$ and $r_{p}$ the particle radius. Associating the above with the effective medium theory and Maxwell’s relation, a relation for the effective thermal conductivity $k_{pe}$ of the effective particles is obtained [20]

(2) $$k_{pe}=\frac{2\left(1-\gamma \right)+{\left(1+\beta \right)}^{3}\left(1+2\gamma \right)}{\left(\gamma -1\right)+{\left(1+\beta \right)}^{3}\left(1+2\gamma \right)}\gamma k_{p},$$

where $\gamma =k_{l}/k_{p}$ is the ratio of the thermal conductivity of the nanolayer to that of the nanoparticle. Eventually, the thermal conductivity of the nanofluid will be

(3) $$k_{eff}=\frac{k_{pe}+2k_{f}+2\left(k_{pe}-k_{f}\right){\left(1+\beta \right)}^{3}f_{p}}{k_{pe}+2k_{f}-2\left(k_{pe}-k_{f}\right){\left(1+\beta \right)}^{3}f_{p}}k_{f}.$$

This model includes the effect of the nanolayer and suggests that very thin nanolayers significantly affect the thermal conductivity of nanofluids, especially when the particle diameter is less than 10 nm. The thickness δ is considered to be between 0–2 nm. Subsequent research has focused on determining the thickness of the nanolayer and its thermal conductivity [21,22,23].

In addition, the use of surfactants is very common when creating nanofluids [24,25]. Surfactants change the interfacial tension between a liquid and a solid and the surface tension between two liquids. Surfactants have been used in a wide range of applications, such as pharmaceuticals, crystal growth and detergents, among others, due to their increased spreading and wetting capability [26]. Recent studies [27,28] examine the rheological and thermal properties of water-based solutions varying the type and the amount of surfactant. It is pointed out that each surfactant affects the properties of the base fluid differently. Moreover, surfactants seem to be a key factor in the preparation and modification of nanofluids. They are adsorbed on the interface between the base fluid and the nanoparticles, and prevent the formation of large aggregates that would precipitate. However, most studies focusing on the changes in thermophysical properties omit the effect of surfactants. Recent experimental studies [24,29] show a maximum value on the effective thermal conductivity for a certain level of the surfactant concentration. Beyond that, the thermal conductivity decreases significantly. Zhai et al. [30] detected different morphology of the aggregates by changing the amount and the type of the surfactant. They pointed out that large aggregates will sediment and they found an optimal volume fraction of surfactants for stable and high conductive Al_2_O_3_/ethylene glycol nanofluid.

In this paper, an algorithm for modelling particle aggregate formation is developed and encoded. This method allows the reconstruction of aggregate systems with predefined characteristics, such as the number of particles in the aggregate, and the fractal dimension. The results are compared with images from an electron microscope, through several criteria in addition to visual resemblance, mainly the value of the surface porosity and the recovery of the correlation function as extracted from the image. In addition, a method for the modelling the effects of the nanolayer and the surfactants is presented. The Meshless Local Petrov–Galerkin (MLPG) method [31,32,33] is used for the solution of the heat transfer equation throughout the working domain and for the calculation of the effective conductivity. Approaches to field functions and their derivatives are made using the Discretisation-Corrected Particle Strength Exchange (DC PSE) method [34], which has been shown to provide stable and fast solutions to such problems, and the integration is performed in cubic sectors around each node. The numerical method that was developed [35] for calculating temperature distribution and effective conductivity can be extended to calculations for a three-phase system, like the one encountered here.

The effective conductivity of the nanofluids as predicted by the present method is compared to the effective conductivity of corresponding systems resulting from known aggregation modelling methods. The comparison reveals a satisfactory agreement as discussed in detail. The effect of particle aggregation on the thermal conductivity is studied by changing the number of particles in the aggregate and the fractal dimension of the aggregates. Also, the effect of the nanolayer and the surfactants on the effective conductivity is examined, and the results are compared with predictions of analytic expressions from the literature.

## 2. Aggregates and Nanolayer Modelling

Particle aggregates may result from a number of forces. The exact mechanism is unknown, and similarly undetermined is the effect of various additional surfactants on aggregation. The effect of temperature on the morphology of the aggregates is also unclear. In studies conducted thus far [10,19], the morphology of the aggregates is considered to have the typical characteristics of particle–particle, particle–aggregate, and aggregate–aggregate clustering. Thus, descriptive models such as Diffusion-limited Aggregation (DLA), Diffusion-limited Cluster Cluster Aggregation (DLCCA), Reaction-limited Aggregation (RLA), and Ballistic Aggregation (BA) are widely used to describe the process of aggregation for various suspended nanoparticles, and the morphology of the nanofluid [35,36]. The majority of the established aggregation models mostly study spherical particles, and this particle geometry was used in the present work as a reference base. However, a variety of non-spherical particles has also been reported in the literature [37]. The fractal character can be quantified through the calculation of the radius of gyration. The radius of gyration of a body is defined as the radial distance from the centre of mass at which, if the total mass of the body is considered to be concentrated, the moment of its inertia would be the same as the real one. For a system of Ν particles with the same mass, the above definition is expressed as

(4) $$R_{g}=\sqrt{\frac{\sum_{i}{\left(r_{i}-r_{c}\right)}^{2}}{Ν}},$$

where $r_{i}\;$ is the position vector of the centre of particle i, and $r_{c}$ is the position vector of the centre of mass. As mentioned above, the fractal dimension, $d_{f}$, is used as a measure for the morphology of aggregates [10,38]. Essentially, it is a measure that indicates how the aggregation spreads in space. The higher its value, the denser the aggregate. Of course, the maximum value can be equal to the working spatial dimensions (2 in 2D, 3 in 3D). The fractal dimension correlates the number of particles in the aggregate with its radius of gyration.

(5) $$N=k_{g}{\left(R_{g}/r_{p}\right)}^{d_{f}},$$

where $k_{g}$ is the structure factor. The linear-fitted slope of the points (N,$R_{g}/r_{p}$) on a double logarithmic diagram determines the fractal dimension, and the point of intersection with the y-axis provides the logarithm of the structure factor. The structure factor is dependent on the connectivity of the particles and the dispersion of the particle size. For monodispersed particles the structure factor can be considered constant, $k_{g}\;=1.5$ [39], whereas $k_{g}$ tends to unity in the limiting case of infinitely polydispersed particles, independently of the agglomeration mechanism [40].

In the DLA model, one particle acts as a core for aggregation, and the rest travel in space by random motion until they come in contact with the aggregate or diverge sufficiently. In the DLCCA model the particles are randomly placed in space, and random motion occurs. As soon as two particles come in contact, a bond is formed. An important limitation is that immobilisation always takes place at the first contact of the particles. The Reaction-limited Cluster Aggregation (RLCA) model introduces a probability that determines how often collisions will form bonds. Another probability, which will determine if a collision causes a particle or a part of the aggregate to detach from the aggregate, can also be defined. This is naturally justified in the case of nanofluids, where there is an additional repulsive force due to the surfactants and the surface charge, which the particles must overcome before they are affected by the short-range van der Waals attractive interactions. 

In the work of Meakin and Joulien [39] it is shown that although the process determines the fractal dimension, the fractal dimension cannot determine the process. For example, aggregates of the same fractal dimension as the RLCA method are formed by using the DLCCA method and allowing a restructuring of the particles when they come in contact. Moreover, structures with large deviations in the fractal dimension can be formed by changing the probability of attachment or detachment [39].

Aiming to study the effect of aggregation and the morphology of aggregates on the thermal conductivity of nanofluids, a technique has been developed and is presented here, where the fractal dimension is predetermined and drives the formation of aggregates. Thus, the addition of particles is performed in a way that meets these fractal dimension requirements. More specifically, the desired fractal dimension, or a range of values for it, is initially determined. Then, permissible values for the number of particles per aggregate are determined and, finally, the volume fraction of nanoparticles and the number of aggregates are defined.

**Figure 1:**
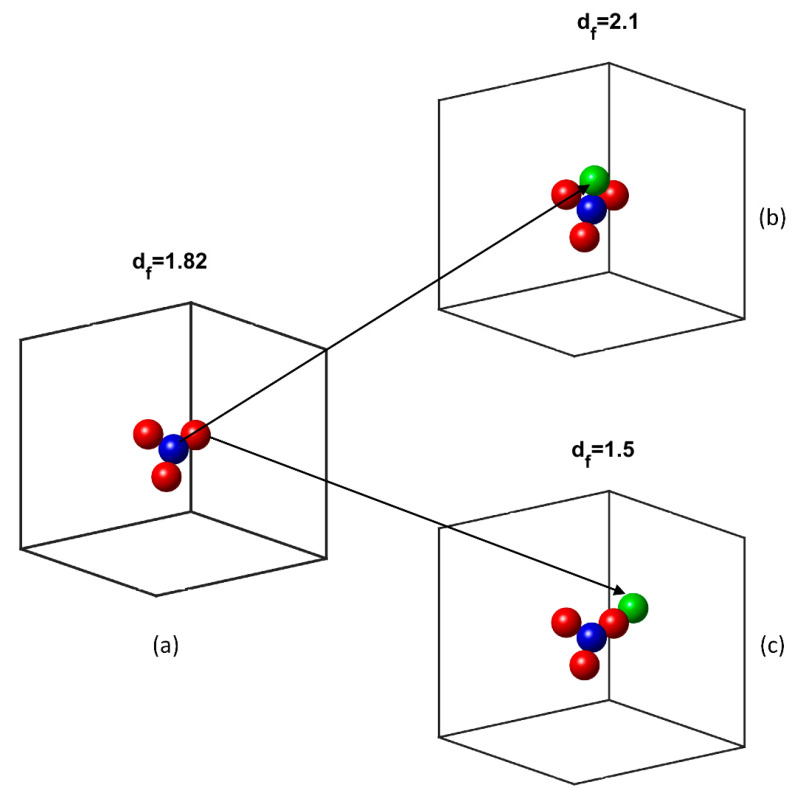
(**a**) Categorisation of particles into internal (blue) and external (red); addition of a new particle (green), which (**b**) increases the fractal dimension and (**c**) reduces the fractal dimension.

**Figure 2:**
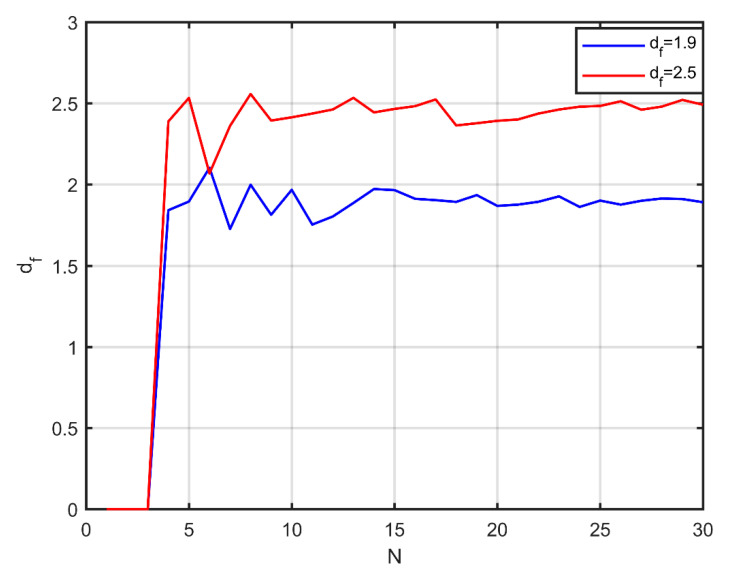
Evolution of the fractal dimension during aggregate formation using the proposed method, for two different fractal dimension values.

The process initiates with a random deposition of a core particle in space. Then, two directional angles are stochastically selected (θ∈[0, 2π],φ∈[0, π]), which determine the point on the surface of the original particle in a local spherical coordinate system, where contact with the second will be made. In general, each subsequent particle appears on the surface of a particle of the aggregate, under the condition of no overlap with any other particle. The process is repeated for a third particle, and then the radius of gyration of the aggregate and its fractal dimension are calculated. The particles that form the aggregate are divided into two categories, namely, those in which the centre lies within the radius of gyration, and those in which the centre lies beyond this radius, as shown in Figure 1a (blue-coloured are the particles within the radius of gyration, red-coloured the particles beyond the radius). Depending on whether the desired fractal dimension is less or greater than the one calculated, the next particle will appear on the surface of a randomly selected particle of the corresponding category. Figure 1b shows the addition of a new particle (in green) that reduces the fractal dimension, while Figure 1c shows the addition of a new particle (in green) that increases the fractal dimension. After calculating the resulting fractal dimension, it is examined whether it approached the predefined value. If not, the added particle is deleted, otherwise the particle remains in place. A new particle appears in the same fashion and the process continues until the aggregate has the desired number of particles. Then, the next particle is deposited at another random point in space, which acts as a core for a new aggregate, and the process is repeated, with the limitation that aggregates do not overlap. This method can stabilise the fractal dimension even in a very small number of particles, as shown in Figure 2.

This methodology, in addition to achieving the desired fractal dimension, has the advantage of being straightforward and fast for calculations. Table 1 shows the comparison of the time required to build two particle systems with the DLA method and with the present method. The first case involves an aggregate containing 30 particles, while the second one involves a system of aggregates containing 300 particles. The superiority of the method in the aggregate formation time is evident, as it can produce structures in less than 1% of the time required by the DLA method.

**Figure 3:**
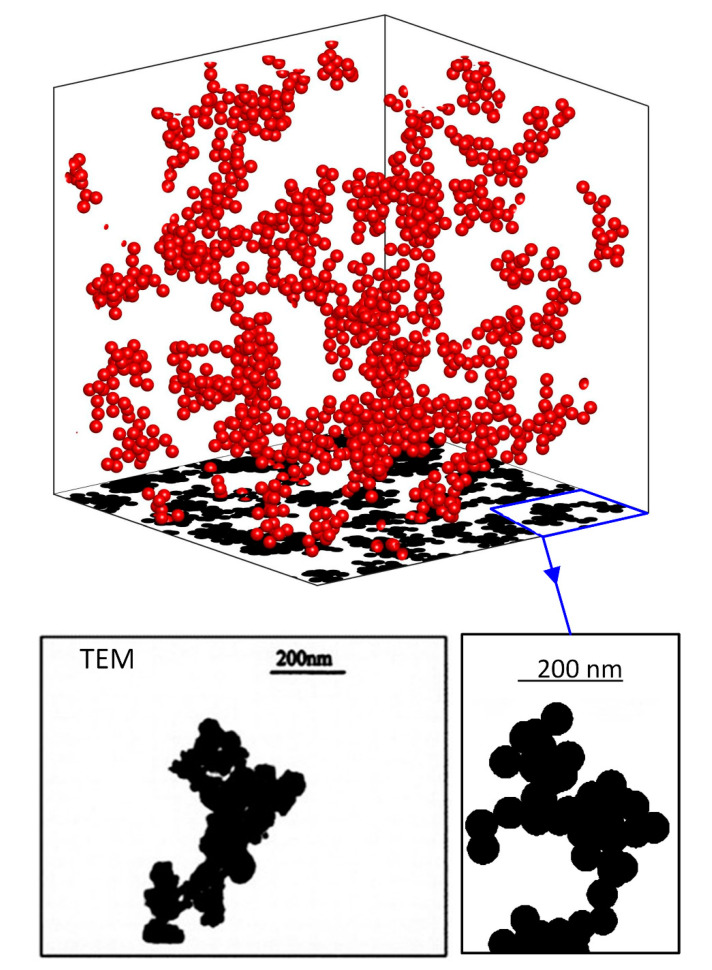
Upper: Simulation example with parameters from experimental data and projection on a 2D plane. Bottom: Comparison of the resulting image with an image from an electron microscope (TEM) [10] following thresholding.

**Figure 4:**
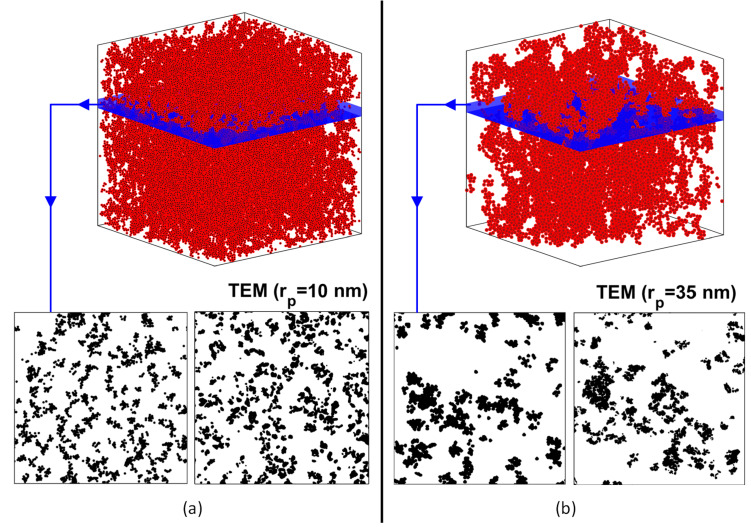
Upper: Simulation examples. Bottom: Comparison of the results with images from an electron microscope (TEM) [41] following thresholding. (**a**) Nickel particles with radius of 10 nm (**b**) nickel particles with radius of 35 nm.

Figure 3 shows a system of nanoparticle aggregates, which includes 50 aggregates consisting of 10 to 18 particles. Their fractal dimension, $d_{f}$, ranges between 1.8 and 2.2., and the volume fraction is $f_{p}=0.02$. Figure 3 also shows an image from electron microscopy (TEM) of a nanofluid consisting of copper particles in water with the same characteristics [10]. The thresholding technique is used to create binary images from the original TEM images to simulate projection onto a plane. Respectively, in Figure 4, images from an electron microscope of two other nanocomposites [41] and the corresponding simulation results are shown for visual comparison. The nanocomposites contain nickel particles with a volume fraction of $f_{p}=0.0574$ for particle diameter 20 nm, and a volume fraction of $f_{p}=0.0552$ for particle diameter 70 nm. Again, the thresholding technique is used to create binary images from the original TEM images in order to be able to compare projected images with simulations. It is observed that in the larger particle case, larger and denser the aggregates are formed, as shown in the TEM images. The fractal dimension of the aggregates has been found to take values from 1.8 to 2.5 [42,43,44]. The depth of the images from the electron microscope is 100 nm.

**Figure 5:**
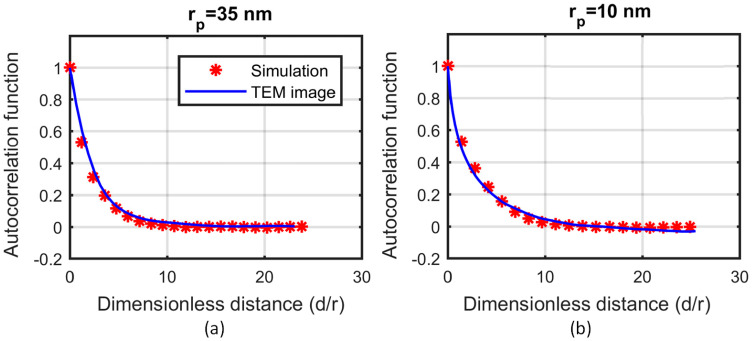
Autocorrelation function of the TEM image (continuous line), and the simulation results (red stars), for (**a**) particle radius 35 nm and (**b**) particle radius 10 nm.

**Figure 6:**
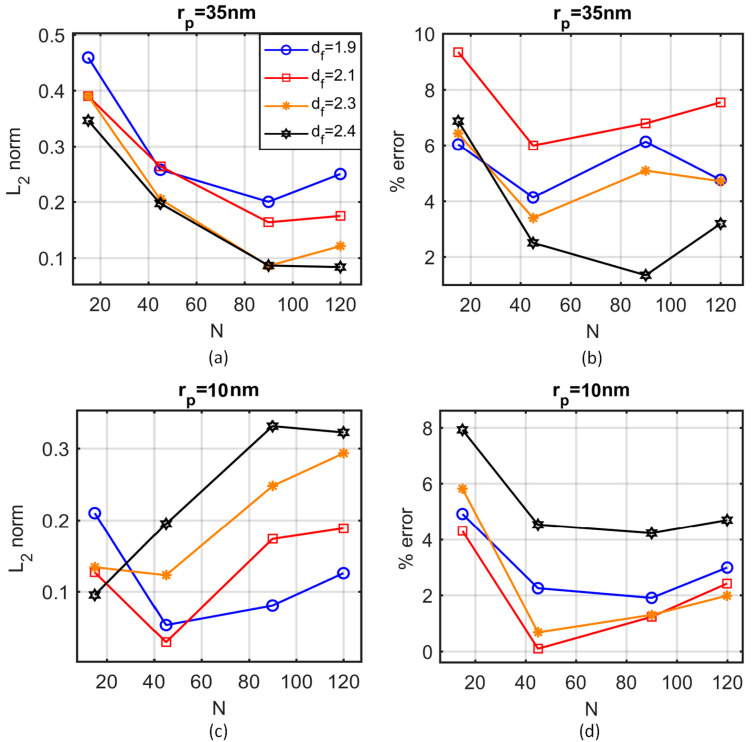
Error in $L_{2}$ norm of the autocorrelation function (**a**,**c**) and the percentage error in the areal porosity (**b**,**d**) as functions of the average number of particles per aggregate, for different values of the fractal dimension, for $r_{p}=35$ nm (**a**,**b**) and $r_{p}=10$ nm (**c**,**d**).

In the absence of accurate information about the fractal dimension or the number of particles in the aggregate, the 2D areal porosity and the autocorrelation function (Figure 5) are computed. Several aggregate systems were constructed by varying the average number of particles (N) and the fractal dimension ($d_{f}$) of the aggregates. A projection of a thin section (100 nm) of the nanofluid on a 2D plane is used to compare the autocorrelation function and the areal porosity of the simulations with those extracted from the TEM images. Figure 6 shows the $L_{2}$ norm of the autocorrelation function (Figure 6a,c) and the percentage error in the areal porosity (Figure 6b,d) for particle radius $r_{p}$ = 10 nm (Figure 6a,b) and $r_{p}$ = 35 nm (Figure 6c,d). The simulation results were derived from the average of 5 simulations, with 10 projected sections each.

Using a specific combination of the morphological characteristics—different for each case—seems to minimise the errors in the areal porosity and the autocorrelation function. In the case of 10 nm, aggregates are selected to consist of 40 to 50 particles, with a fractal dimension between 2.0 and 2.2. Respectively, in the case of 35 nm, the aggregates are selected to consist of 80 to 100 particles, with a fractal dimension between 2.3 and 2.5. Figure 5 shows the autocorrelation function of the simulation results for the aforementioned characteristics and the TEM images. The dimensionless distance is defined as the ratio of the actual distance to the radius of the particle, $r_{p}$. Figure 4 shows the same simulations for the sake of visual comparison with the TEM images.

**Figure 7:**
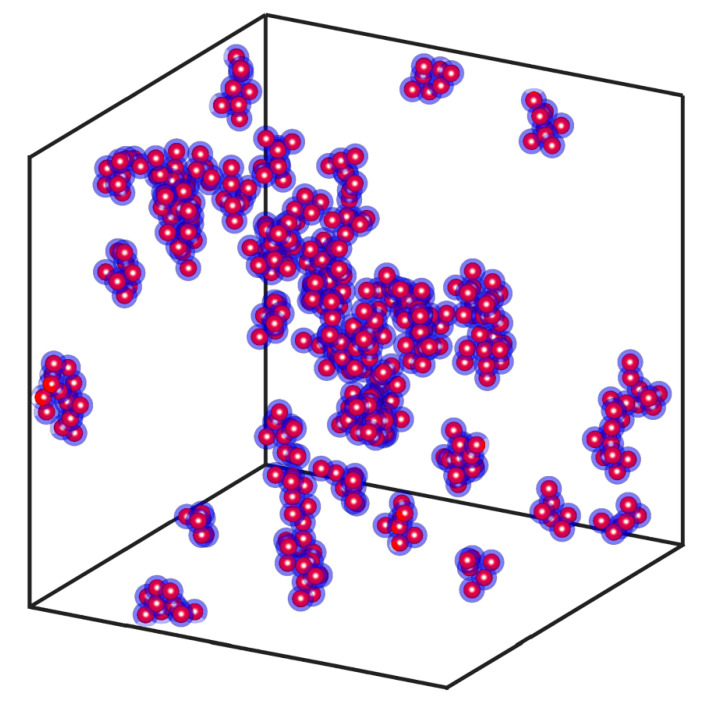
System of nanoparticle aggregates consisting of 5 to 10 nanoparticles with fractal dimension ranging from 2.1 to 2.4, with a volume fraction of 0.01 and a nanolayer thickness of 45% of the particle radius.

For the nanolayer modelling, the process is also straightforward. Considering the radius of the nanoparticles constant, $r_{p}$, a thickness, δ, is defined for the surfactant nanolayer. For fully dispersed particles, the only restriction is that there is to be no overlapping of the particles. In the case of clusters, overlapping cannot be avoided. The nanolayer is tested in order to avoid any overlapping between parts of neighbouring aggregates. Figure 7 shows such a system of nanoparticle clusters, with a volume fraction $f_{p}=0.01$ and a nanolayer thickness of 45% of the particle radius. In this approach, we assume that the surfactant forms a spherical shell around each particle. In reality, it is expected to have a somewhat different distribution, especially in the case of particle aggregates. The thickness of the layer can be calculated by

(6) $$\delta =\sqrt[{3}]{\frac{3\left(f_{p}+f_{l}\right)V}{4\pi N_{P}}}-r_{p}{}^{\;},$$

where $f_{l}$ is the volume fraction of the surfactant, V is the total volume of the working domain and $N_{P}$ is the total number of particles.

## 3. Effective Conductivity Calculation

Heat transfer is caused by a temperature difference that is imposed on the material in a certain direction, in this case, along the vertical axis. Periodic conditions are applied at the other transverse boundaries of the medium. The simulations for the solution of the heat conduction problem are performed with innovative meshless methods, which offer accurate and fast solutions in a wide range of conditions. In fact, in the particle aggregate case, the lack of mesh allows easy local increase of spatial discretisation at the interface of the nanoparticles and the base fluid, where steep gradients develop. The numerical solution of the energy equation is performed using the Meshless Local Petrov--Galerkin (MLPG) method [33]. This method is based on the expression of partial differential equations in their local weak form, i.e., their integration in local sub-sectors. The approach to variables and derivatives is achieved with the DC PSE method [34]. All integrals are calculated in cubic sectors around each node, as this has been shown to increase the stability of the method [32,35], and square grids are used with increased resolution in the area around the interfaces. A complete analysis of the mesh construction and the approach to variables and integrals is given in [35]. The dimensionless weighted integral form of the heat equation in the $\Omega_{x}$ sector is given by the relation

(7) $$\int_{\Omega_{x}}^{\;}\nabla \left(k\nabla T\right)vd\Omega =0,$$

where v is the weight function of integration (here, the step function), and k=k(x) is the thermal conductivity. Two step functions $\Phi_{p},\Phi_{l}$, are defined, with values equal to unity in the particle area ($\Phi_{p}$) or the layer area $\left(\Phi_{l}\right)$, and zero elsewhere. Using the deviation theory, the following weak form of the energy equation is obtained

(8) $$\int_{\partial \Omega_{x}}^{\;}\left(k_{r}{}_{p}-1\right)\Phi_{p}\nabla T_{\hat{n}}d\left(\partial \Omega_{x}\right)+\int_{\partial \Omega_{x}}^{\;}\left(k_{r}{}_{l}-1\right)\Phi_{l}\nabla T_{\hat{n}}d\left(\partial \Omega_{x}\right)+\int_{\partial \Omega_{x}}^{\;}\nabla T_{\hat{n}}d\left(\partial \Omega_{x}\right),$$

where $k_{r}{}_{p}=\frac{k_{p}}{k_{f}}$ is the ratio of the thermal conductivity of the particles to that of the base fluid, and $k_{r}{}_{l}=\frac{k_{l}}{k_{f}}$ is the ratio of the thermal conductivity of the nanolayer to that of the base fluid. Solving the above equation offers the temperature distribution throughout the domain space. It is, thus, possible to calculate the effective thermal conductivity of the area from the following integral

(9) $$k_{eff}=\bar{\underset{S}{\int }k\frac{\partial T}{\partial n}dS}.$$

The surfaces S in which the calculations are made are vertical to the heat flow. At least 10 such surfaces equidistantly distributed along the imposed heat gradient axis are used to determine the conductivity, and their mean value determines the effective thermal conductivity. The present method for effective conductivity calculations can be extended to consider different nanoparticle shapes. The check for overlapping must take into account the equations that describe the external surfaces of the nanoparticles. The possible rotation of nanoparticles can also be taken into account, most simply in a random fashion around the centre of mass.

## 4. Results and Discussion

### 4.1. Comparison with Other Aggregation Models

**Figure 8:**
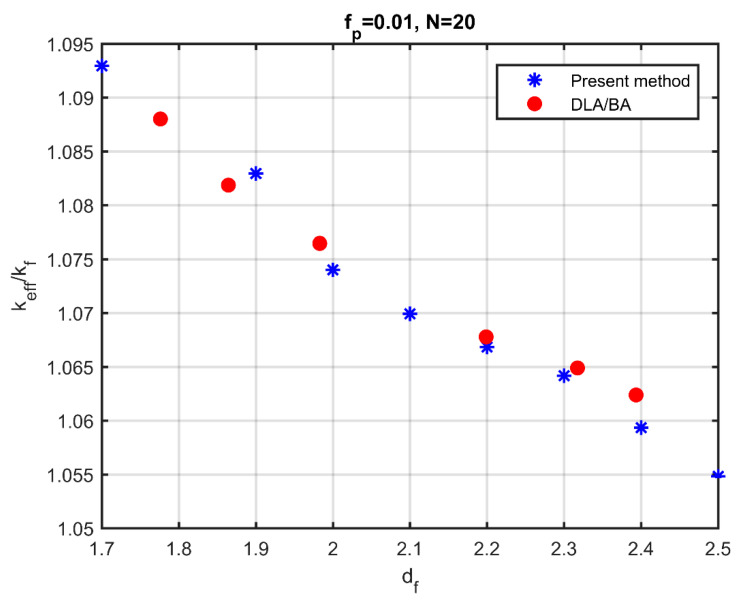
Effective thermal conductivity as a function of the fractal dimension of aggregates by the present method (blue points) and by simulations from [35] (red points). DLA and BA (ballistic) estimates are practically identical.

In an earlier work of the authors [35], the effect of aggregation on the thermal conductivity was studied using aggregates derived from the DLA and the ballistic-type deposition method. It was observed that aggregates with the same fractal dimension and the same volume fraction, resulting from different aggregation methods, had, practically, the same thermal conductivity. Figure 8 shows the comparison of the results of these previous simulations with the results of the aggregation method developed here. The volume fraction of particles is $f_{p}=0.01$, and the particles are organised into aggregates consisting of Ν=20 particles. The conductivity of the particles is considered $k_{r}{}_{p}=\frac{k_{p}}{k_{f}}=\;100$ times larger than that of the base fluid. The simulation points are derived from the mean of 10 simulations with the same characteristics. The results are quite similar, which supports the belief that the mechanism for aggregation is not significant for the effective conductivity in contrast with the significance of the morphological characteristics of the aggregates. It also appears that the present method produces aggregates that are similar to those resulting from methods that describe the physical process of particle aggregation. 

### 4.2. Dependence of Conductivity on the Fractal Dimension and the Number of Particles in the Aggregate

A parametric study of the effect of the fractal dimension and the number of particles in the aggregate is presented next, using the methodology that is developed in the present work. The conductivity of the nanoparticles is chosen to be 130 times greater than that of the base fluid ($k_{r}{}_{p}=130$), which is relevant in several practical nanofluids, like water-Fe, water-CuO, and engine oil-Al_2_O_3_. The simulation points are derived from the average of ten simulations with the same characteristics (volume fraction $f_{p}$, number of particles per aggregate N, fractal dimension $d_{f}$).

**Figure 9:**
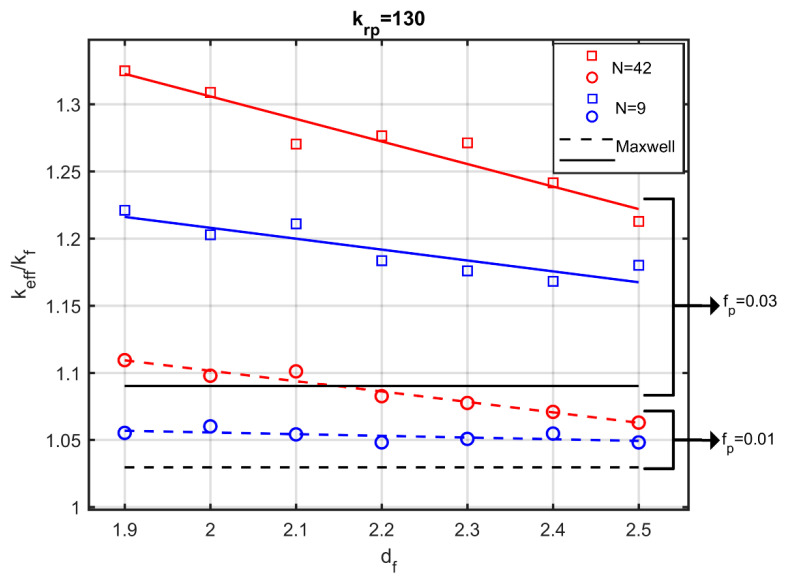
Effective thermal conductivity as a function of fractal dimension. Solid lines: $f_{p}=0.03$. Dashed lines: $f_{p}\;=0.01$. Blue lines: 9 particles per aggregate. Red lines: 42 particles per aggregate. Black lines: Maxwell’s model.

Figure 9 shows the dimensionless effective thermal conductivity as a function of the fractal dimension for two values of the particle volume fraction and for two different numbers of particles per aggregate. Figure 9 also shows the conductivity prediction according to Maxwell’s model. A first remark is that, even for small volume fractions of the particles ($f_{p}=0.03$), a noticeable change of the conductivity is obtained by modifying the fractal dimension, while the effective-medium theory and, specifically, the Maxwell’s relation (Equation (1)), remains insensitive to the fractal dimension.

More specifically, the effective thermal conductivity apparently decreases with increasing fractal dimension. That is, the more cohesive the aggregate becomes, the lower the conductivity of the nanofluid. The rate of reduction is affected by both the volume fraction of the particles and the number of particles that form the aggregate.

**Figure 10:**
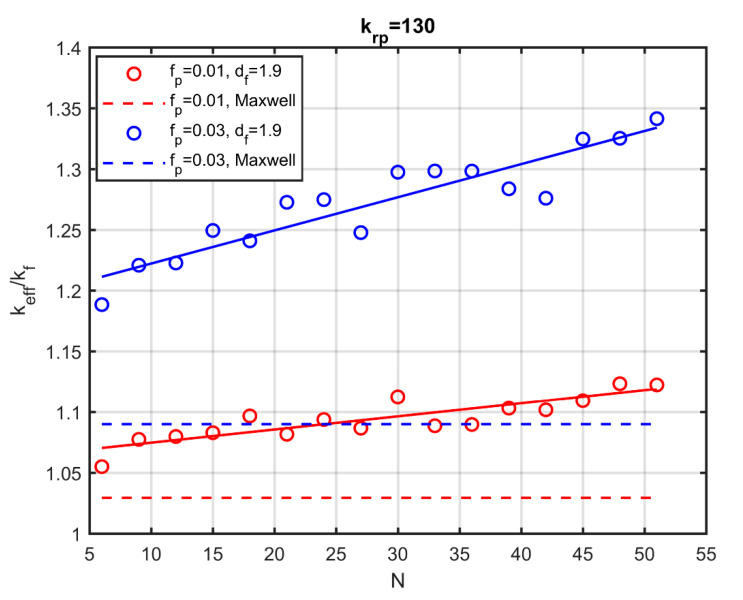
Effective thermal conductivity as a function of the number of particles in the aggregate. Blue lines:$f_{p}=0.03$, Red lines: $f_{p}=0.01$ Dashed lines: Maxwell’s model.

Figure 10 shows the effect on conductivity by changing the number of particles in the aggregate, for two volume fractions of the particles and the same fractal dimension $d_{f}=1.9$. There is a significant increase in conductivity even for aggregates with a small number of particles. When the aggregates consist of six particles, a 20% increase is observed in the conductivity for volume fraction $f_{p}=0.03$ (blue points). The more particles in the aggregate, the greater the increase. For 50 particles in the aggregate, the conductivity of the nanofluid increases by 35%. For this volume fraction, Maxwell’s relation predicts an increase of less than 10% (blue dotted line). 

### 4.3. Dependence of Conductivity on Volume Fraction and Particle Conductivity

**Figure 11:**
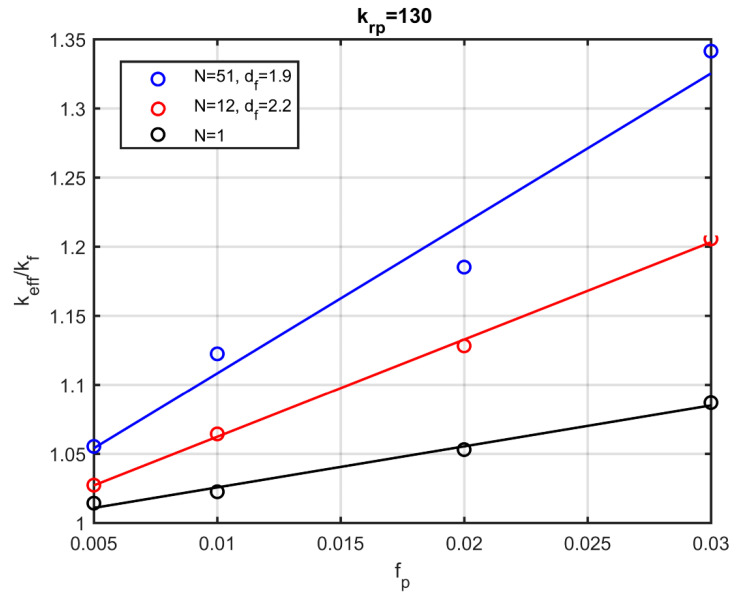
Effective thermal conductivity as a function of the volume fraction of the particles.

Furthermore, the variation in the effective conductivity by increasing the particle volume fraction and the ratio of the conductivity of the particles and the fluid is examined. Figure 11 shows the increase in conductivity with the addition of more particles for three characteristic cases. For fully dispersed particles (Ν=1) the simulation results are identical to those of Maxwell’s model. A substantial increase of 20% for the nanofluid conductivity is observed, even for quite coherent ($d_{f}=2.2$) and small (Ν=12) aggregates.

**Figure 12:**
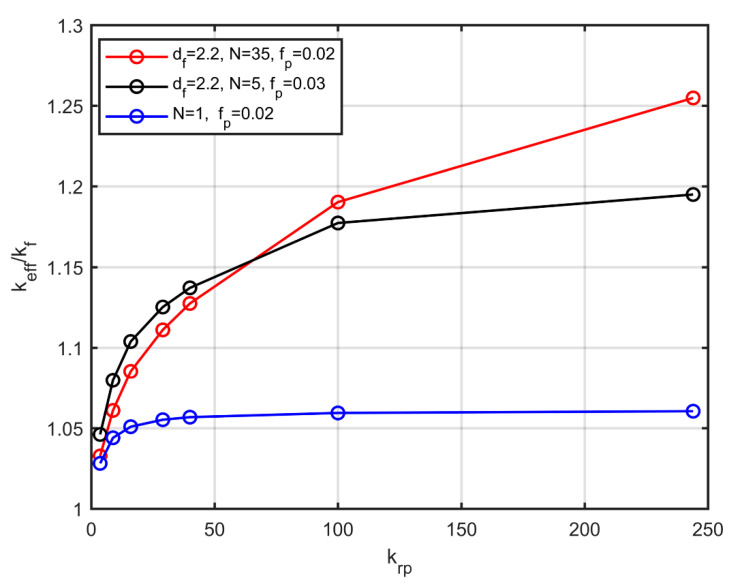
Thermal conductivity as a function of the ratio of the conductivity of the particles and that of the base fluid, for three characteristic cases.

Figure 12 shows the thermal conductivity as a function of the ratio of the conductivity of the particles and that of the base fluid, for three characteristic cases. For fully dispersed particles (Ν=1), the more conductive the particles are, the higher the effective conductivity. Beyond a certain value ($k_{r}{}_{p}\sim \;50$), the nanofluid conductivity appears to become progressively less sensitive to the particle conductivity. On the other hand, for the same volume fraction ($f_{p}=0.02$), with the particles organised into aggregates of Ν=35 particles with fractal dimension $d_{f}=2.2$, the effective conductivity continues to increase monotonically with the increase of the particle conductivity. Furthermore, the case of Ν=5 particles in the aggregate and a volume fraction of $f_{p}=0.03$, is compared with the case of Ν=35 particles and a volume fraction of $f_{p}=0.02$. Both cases have the same fractal dimension, equal to $d_{f}=2.2$. For small conduction ratios, a nanofluid with a larger volume fraction has a higher effective conductivity. On the contrary, for more conductive particles the behaviour changes, and a nanofluid with a lower volume fraction is more conductive. Therefore, with appropriate aggregation one can have greater effective conductivity with a lower volume fraction of particles. This is attributed to the fact that, for the same fractal dimension, larger aggregates transfer heat more efficiently to the fluid, as they create longer conductive paths. 

### 4.4. Effect of Nanolayer/Surfactants

The effect of nanolayer and surfactants is also examined. The nanolayer is expected to have a conductivity between the value of the base fluid and that of the particles. On the other hand, depending on the case, the conductivity of the surfactants can be lower than that of the base fluid.

**Figure 13:**
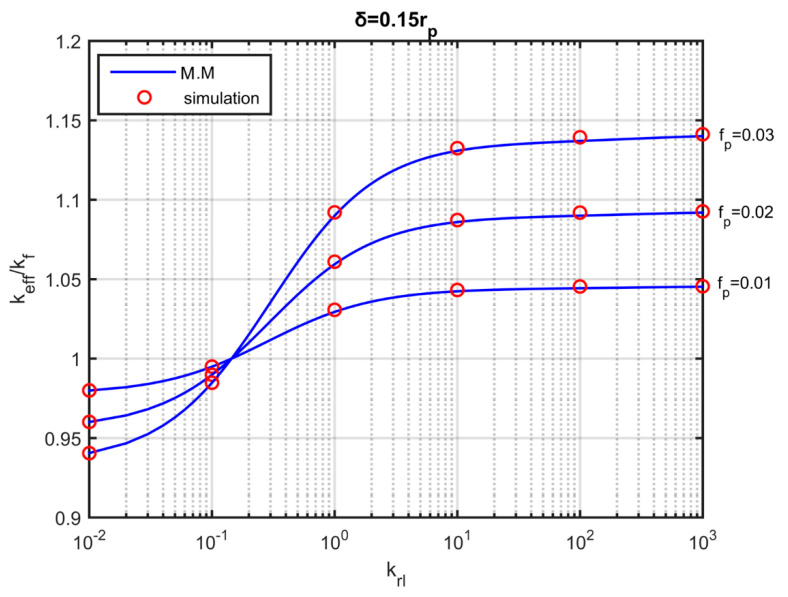
Effective thermal conductivity as a function of the nanolayer conductivity in fully dispersed particles for three volume fractions. Blue lines: results of the modified Maxwell ‘s model (M.M.). Red dots: simulation results.

Figure 13 shows the effective conductivity of a nanofluid that contains fully dispersed particles in the presence of a nanolayer, as a function of the ratio of the nanolayer conductivity to that of the base fluid. The thickness of the nanolayer is assumed to be 15% of the particle radius, and the volume fraction ranges from $f_{p}=0.01$ to $f_{p}=0.03$. The ratio of the particle conductivity to that of the fluid is $k_{r}{}_{p}=100$. Figure 13 also shows the modified Maxwell’s model (M.M.) (Equation (3)) for the calculation of the effective conductivity of nanofluids that takes into account the existence of a nanolayer. The results show that the effective conductivity of the nanofluid is lower than that of the base fluid for nanolayer conductivity $k_{r}{}_{l}<0.15$, and increases dramatically by increasing the nanolayer conductivity up to a certain $k_{r}{}_{l}\;$limit ($k_{r}{}_{l}\;\sim \;10$). The simulations are in good agreement with the analytical model. 

**Figure 14:**
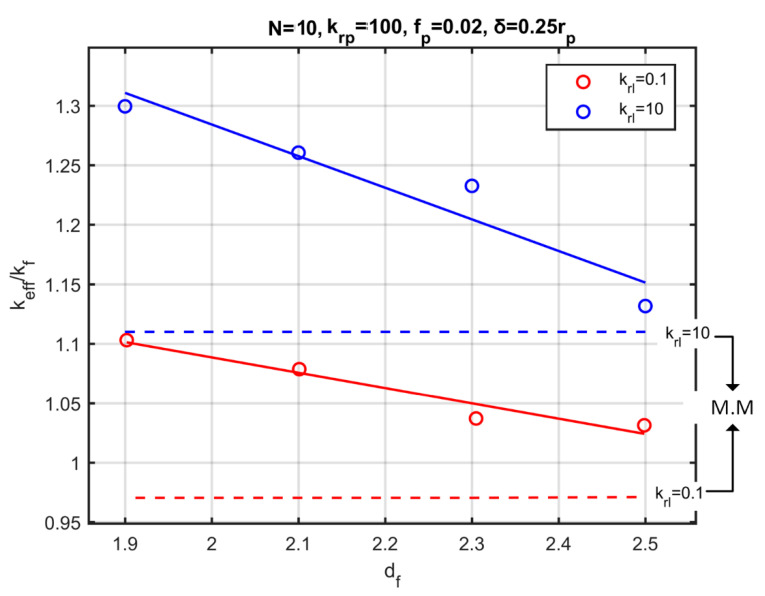
Effective thermal conductivity as a function of the fractal dimension for aggregates with a nanolayer. Blue dots: ratio of the nanolayer conductivity to the base fluid conductivity $k_{r}{}_{l}=10$. Red dots: $k_{r}{}_{l}=0.1$. Dashed lines: Modified Maxwell’s model.

Figure 14 portrays thermal conductivity results for nanoparticles organised into aggregates containing Ν=10 particles each, with volume fraction $f_{p}=0.02$, and nanolayer thickness equal to 15% of the particle radius, varying with the fractal dimension. The conductivity of the nanolayer ranges from $k_{r}{}_{l}=0.1$ to $k_{r}{}_{l}=10$. Figure 14 also shows the modified Maxwell’s model for these cases, and depicts its significant deviation, since it does not take into account the effect of aggregation. As in the case without a nanolayer, small values of the fractal dimension enhance nanofluid conductivity substantially. In the presence of a conductive nanolayer, and with Ν=10 particles in each aggregate, a ~30% increase is obtained, as shown in Figure 14. In the absence of the nanolayer and for the same aggregates (Figure 8), the increase is ~15%. Furthermore, the presence of aggregates outweighs the decrease in conductivity due to low nanolayer conductivity, noted in Figure 13. That is, even for a less conductive nanolayer compared to the base fluid, the resulting nanofluids have increased effective thermal conductivity.

### 4.5. Comparison with Experimental Data

**Figure 15:**
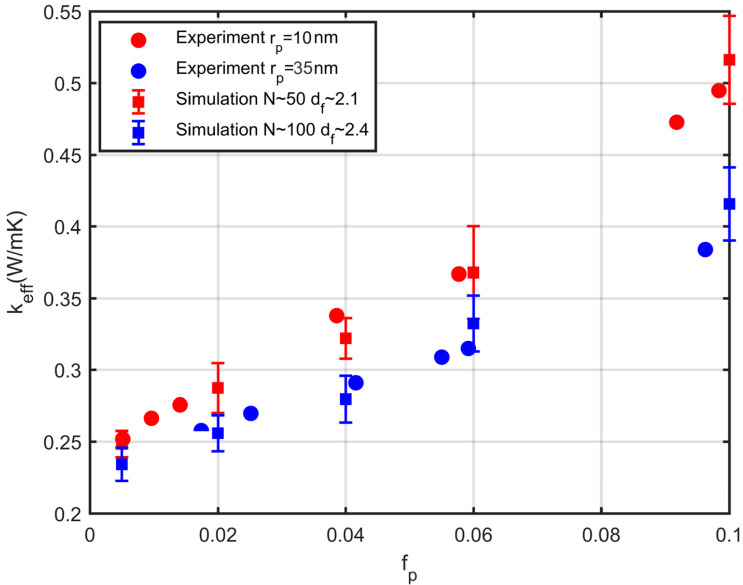
Comparison of experimental results [41] (dots) and simulation results (squares). Red dots, particles of radius $r_{p}=10\;\mathrm{nm}$, red squares, simulation of N~50 particles per aggregate and fractal dimension,$\;d_{f}\sim 2.1$, blue dots, particles of radius $r_{p}=35\;\mathrm{nm}$, blue squares, simulation of N~100 particles per aggregate and fractal dimension,$\;d_{f}\sim 2.4$.

The predictions of the effective conductivity for the nanofluids that were studied above are compared in this section with experimental measurements from the literature. In the context of work [41], an epoxy nanocomposite material was formulated, containing nickel nanoparticles (Ni). A number of such samples were created by modifying the diameter of the nickel particles and their thermal conductivity was measured. The results are shown in Figure 15, along with the results of our simulations. The simulation points are derived from 10 simulations with the same characteristics, and the standard deviation of the results is shown in Figure 15. The results of the simulations are in good agreement with the experimental data.

**Figure 16:**
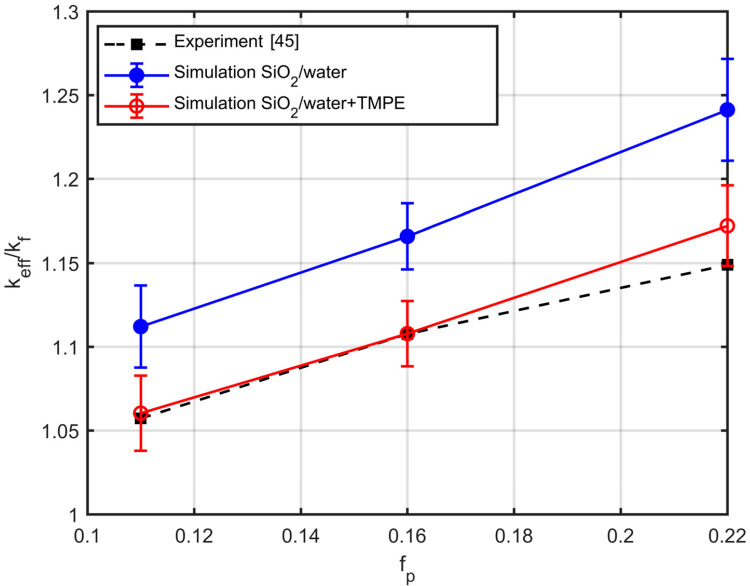
Comparison of experimental results, extracted from [45] (black squares) and simulation results, without the surfactant (blue dots) and with the surfactant (red dots).

Figure 16 compares simulations and experimental measurements for SiO_2_ nanoparticles dispersed into water for various volume fractions. Their average radius of gyration and effective conductivity were measured in the literature [45]. The aggregates consist of approximately Ν=7 particles, and their fractal dimension is approximately $d_{f}=1.9$ [19]. As shown in Figure 16, the effective conductivity resulting from the simulations shows a constant deviation from the experimental data. This can be attributed to the fact that, for the stabilisation of the nanofluid, the authors of [45] used the silane N-[3-(trimethoxysilyl)propyl]ethylenediamine (TMPE), with a w/w concentration of 0.05 g/mL. This is equivalent to a volume fraction of $f_{l}=0.05$. Thus, one can estimate the thickness of the nanolayer from Equation (6), which ranges within 7–16% of the radius of the particles. The thermal conductivity of silane is about 1/3 of the conductivity of water, while the conductivity of SiO_2_ is 2 times greater than that of water. The thermal conductivity of the materials is shown in Table 2. Taking this into account, the agreement between simulations and experiment improves drastically, as shown in Figure 16.

**Figure 17:**
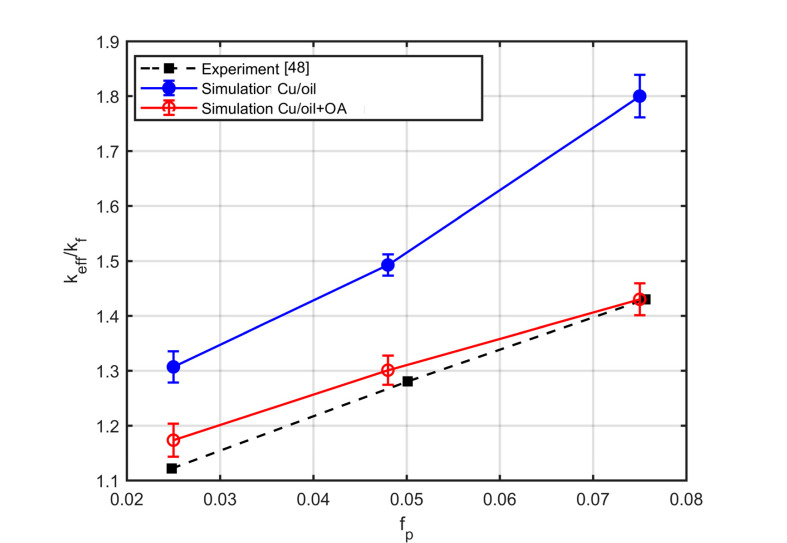
Comparison of experimental results [48] (black squares) and simulation results, without the surfactant (blue dots) and with the surfactant (red dots).

Similarly, in the context of work [48], copper nanoparticles are dispersed into mineral oil, and the nanofluid is stabilised by the addition of oleic acid (O.A). The volume fraction of the oleic acid changes as follows, $f_{l}=2.2f_{p}$, where $f_{p}$ is volume fraction of copper particles. The particles are reported [10] to be organised into aggregates of 14 particles, with a fractal dimension of $d_{f}=2$. Figure 17 shows the experimental measurements and the results of the simulations with two options, namely, accounting for the layer of the surfactant or ignoring it. The deviation of the simulations from the experiment is about 20% when ignoring the nanolayer and is reduced to less than 3% when the nanolayer is included in the simulations.

## 5. Conclusions

In the present paper, the effect of the aggregation of nanoparticles on the thermal conductivity of nanofluids was studied, accounting also for the role of surfactants. To this end, a method for reconstructing particle aggregates is proposed, with desired morphological characteristics. The fractal dimension and the number of particles in the aggregate are predetermined and eventually matched by the resulting aggregates. In order to ensure successful reconstruction, the morphological characteristics for the nanofluids are chosen through the minimisation of the deviation in the autocorrelation function and the areal porosity of the simulations from those that are extracted from the electron microscope images. The method is also extended to include a nanolayer around the particles, simulating the presence of surfactants. The numerical method that is used here for the solution of the conduction equation allows for fast calculation of the thermal conductivity in large two-phase and three-phase nanoparticle systems, practically without any instability problems.

The nature of the surfactant affects aggregation through the intermolecular forces that are developed at the particle interfaces. Consideration of such effects at the molecular level would require a force field description at a totally different scale of simulation and would, inevitably, increase aggregation simulation by orders of magnitude. The proposed model uses as input some readily measurable quantities, like the fractal dimension, the number density of the aggregates and the amount of surfactant and converges quickly.

The results of the method were compared with ones from other aggregation methods, and a good agreement was obtained. In addition to its simplicity in implementation, the new method proves much faster than other aggregation models that simulate the aggregation process physically. The change in the effective conductivity was studied by changing the fractal dimension of the aggregates and the number of particles in the aggregate. It has been shown that aggregation, even with a small number of particles, leads to a notable increase in the effective conductivity. The fractal dimension and the number of particles in the aggregate were found to play key roles in the thermal behaviour of the nanofluids, since significant differences are observed upon their variation. Considerable increase in conductivity occurs for small values of the fractal dimension and large numbers of particles in the aggregate.

The effective thermal conductivity is significantly affected by the nanolayer conductivity. Maxwell’s modified model (M.M) for calculating effective conductivity, which takes into account the existence of a nanolayer, is in very good agreement with simulation results for fully dispersed particles only, for all the particle volume fractions studied here. However, the M.M model fails considerably as soon as the particles are organised into aggregates, since it does not take into account the effect of aggregation. The simulations are in satisfactory agreement with experimental results. It is shown that knowledge of the type and the amount of the surfactants used is required to predict the conductivity of the nanofluids accurately. Simulations that ignore the existence of surfactants may lead to large deviations of the nanofluid conductivity from experimental data. 

## Figures and Tables

**Table 1:** Time required to construct an aggregate and an aggregate system, with the Diffusion-limited Aggregation (DLA) method and with the present method.

Number of Particles/Aggregate(s)	DLA	Present Method
30 particles/single aggregate	25 s	0.2 s
300 particles/aggregate system	303 s	1.3 s

**Table 2:** Thermal conductivity of the component materials [41,45,46,47].

	Ni/epoxy (W/(mK))	SiO_2_/water+TMPE (W/(mK))	Cu/oil+O.A (W/(mK))
Nanoparticles	90	1.43	400
Base fluid	0.21	0.7	0.37
Surfactant	-	0.25	0.2
